# Auscultation in Boston in 1793

**Published:** 1858-07

**Authors:** 


*Auscultation in Boston in* 1793.—To talk about auscultation as employed for the diagnosis of disease during the last century, will perhaps be received with a smile of incredulity; and yet whoever will consult the Memoirs of the American Academy of Arts and Sciences, Vol. II., Part I., 1793, will find a case in which the application of the ear to the chest of the patient enabled the physician to diagnosticate the fact of a communication of an abscess in the thoracic walls with the lung. It is interesting to observe that the individual who came so near immortalizing himself by the discovery of auscultation, was the celebrated Dr. Edward Augustus Holyoke, or *Master* Holyoke, as his pupils still delight to call him, who was no less distinguished for his scientific attainments, and professional skill, than for the fact that he lived upwards of one hundred years in the full possession of his faculties. The following outline of the case is taken from the Memoirs of the Academy, to which it was communicated by Dr. Holyoke.

The patient was a man of about 53 or 54 years of age, of a thin habit of body, with a very bad cough, hectic fever, profuse sweats, &c. He had a large tumor, of about the breadth of the hand, below the left clavicle, extending from the shoulder to the sternum. This tumor had all the appearance of an abscess, and was treated as such. Suppuration appeared to be coming on, when, one day, it appeared less prominent than usual, and was flabby to the touch, while the pain and inflammation had abated. The physician was at a loss what to make of the case, when the patient asked, "what could occasion that blubbering noise in the sore?" "On applying my ear to the part," says Dr. Holyoke, "I plainly heard a whizzing, and, as he termed it, a blubbering noise, at every breath, exactly resembling such as arises from the rushing of air through a small orifice. This orifice appeared to be just under the left clavicle, but nearer to the shoulder than the sternum. Upon viewing the part attentively, a small dilatation and contraction was perceptible upon expiration and inspiration, and the part was evidently puffy and flatulent to the touch. At this time the cough was very urgent, and the expectoration very copious." The swelling, inflammation and hardness subsided; the noise in breathing gradually lessened till it ceased, the cough, hectic and sweats left him, the appetite and strength slowly returned, and the patient was in tolerable health when the case was reported.

Dr. Holyoke's opinion was, that the abscess formed in the thoracic parietes originally, and afterward penetrated to the lung, which came adherent to the walls at this part, and discharged itself through the bronchi. The abscess having a communication with a cavity in the lung, air from the latter would pass into it with every expiration, and be drawn back again with every inspiration; "and this passing and re-passing of the air," continues Dr. Holyoke, "will fully account for the noise which the patient complained of."

Taking into consideration the emaciation, cough and hectic fever, it seems probable that the case was one of empyema, from pleuritic inflammation, in which the matter pointed outwardly, but before discharging through the skin, burst into the lung, and was evacuated through the bronchi. The pathology of thoracic diseases being less perfectly understood at that time than at present, it is not surprising that Dr. Holyoke should have supposed the abscess to have formed externally to the pleuritic cavity, and to have afterward made its way into the lung. But however the fact may have been, the case is one of great interest, as showing how near a person of more than common sagacity may approach to a great truth without discovering it.— This is not the first instance in which the great discovery of Laennec was almost anticipated. Dr. Walshe has happily chosen as a motto for his work on the "Diseases of the Lungs, Heart and Aorta," a quotation from R. Hook, written in 1705—"Who knows but that we may discover the works performed in the several offices and shops of a man's body by the sounds they make, and thereby discover what instrument and engine is out of order?" Had Dr. Holyoke thought of applying the knowledge he obtained in this case to the diagnosis of thoracic diseases in general, his name would have gone down to posterity as one of the most illustrious in the annals of medicine.

We copy the above from the Boston Journal, as showing how we have had great discoveries almost within our grasp, yet the mind has not followed out the path which seems now to have been almost clear, and years have passed, reserving the glory for another. We have often looked with wonder upon the quotation of Hook, which is referred to in this article, marvelling that none had attempted, before the time of Laennec, to "discover the works performed in the several offices and shops of a man's body by the sounds they make."

